# The Impact of an Educational Program on Blood and Blood Products Transfusion on Nurses’ Level of Knowledge and Performance

**DOI:** 10.25122/jml-2018-0016

**Published:** 2018

**Authors:** Mohammad Islami Vaghar

**Affiliations:** 1. Department of midwifery, Tehran Medical sciences branch, Islamic Azad university, Tehran, Iran

**Keywords:** Knowledge and Performance, Education, Transfusion, Nurses

## Abstract

**Objective and background:** This study aimed to evaluate the impact of an educational program on blood and blood products transfusion on nurses’ knowledge and performance in hospitals affiliated with Mashhad University of Medical Sciences.

**Method:** The present study was a semi-experimental before-after study. We selected study participants through the simple random sampling method using sampling framework of all reference population. Data collection tool was a valid and reliable questionnaire. In order to evaluate the effect of the educational program on nurses’ level of knowledge and practice, we asked them to fill out the questionnaires before and after the education course. We analyzed data using SPSS 22. Descriptive statistics such as frequency and percentages and analytical statistics such as Mann- Whitney, Wilcoxon and Kruskal-Wallis were used to report the results. The significance level of the p-value was assumed to be < 0.05.

**Results:** Our analysis revealed a significant difference in the mean score of the nurses’ level of knowledge before and after the education (p=0.001). There was also a significant difference in the mean score of veteran nurses’ performance before and after the intervention (p<0.05). The association between sex and mean score of nurses’ performance was not significant before and after the intervention. The association between nurses’ knowledge score and age, sex, work experience and department of the hospital was not significant (p>0.05).

**Conclusion:** Educational programs about blood transfusion can positively influence nurses’ knowledge and performance. There is a need to improve nurses’ knowledge and performance about the inadvertent side effects of blood transfusion.

## Introduction

Millions of patients need blood and blood products transfusions all around the world [1]. Blood transfusion is defined as the process by which the blood of one person is injected into another one’s circulation for medical purposes. In the early twenty century, blood transfusion contributed to various adverse side effects; however, nowadays these effects are preventable through educating medical care providers and screening blood and blood products [2]. Blood transfusion is a vital need for some patients, but without caution, it can be a life-threatening intervention [3, 4]. It is estimated that in every 13000 cases of blood transfusion one error occurs mostly due to human errors which are preventable through appropriate education and reform in blood transfusion protocols [5, 6]. Regarding the important role of nurses in a safe and effective blood transfusion, it is necessary to improve their knowledge and skills [1]. Blood transfusion is a complex procedure including five stages and nurses are involved in four of them including preparing blood units, collecting blood packs, activities related to before and after transfusion and patient safety monitoring [7,8]. Blood transfusion is an important medical intervention which requires sufficient knowledge and skills [2]. Many studies have been conducted on the level of knowledge and awareness of nurses and physicians about blood transfusion [1,3,7]. However, regarding the increasing demand for blood transfusion in hospitals and its role in saving patients’ lives, it seems necessary to improve nurses’ level of knowledge and performance to ensure the safety of this intervention. This study aimed to evaluate the impact of educational programs on blood and blood products transfusion on nurses’ level of knowledge and skills in hospitals affiliated with Mashhad University of Medical Sciences.

## Methods

We conducted a semi-experimental before-after study on nurses in hospitals affiliated with Mashhad University of Medical Sciences in 1396. The sample size was estimated to be 25 participants based on previous studies [9], using: 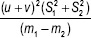 Formula, significance level of 0.05 and the power of study equal to 0.9. We included 50 participants to improve the power of the study. We used the simple random sampling method to collect study participants. Each nurse in different departments of the hospital had the same probability to be included in the study. Inclusion criteria were: having at least a B.Sc. degree, at least two years of working experience in hospital departments related to blood and blood products transfusion and not having attended any educational courses about transfusion before. Exclusion criteria were: not attending the course due to illness, traveling or being reluctant to participate in the study, being absent for more than one session. Data collection tool was a questionnaire designed by Pourfarzad and et al. which was validated under the supervision of prominent professors in this field [10] This questionnaire had three parts: the first part consisted of demographic information (age, sex, level of education, marital status, working experience, hospital department). The second part included 14 questions regarding the knowledge area such as the allowed interval of injecting one unit of blood, the allowed interval between taking out blood packs from the blood bank until the injection, the allowed serums that can be prescribed at the same time with the blood serum, the names of coagulation factors, the adverse side effects of blood transfusion, the appropriate reactions to hemolytic complications, the rational use of platelet transfusion, hemoglobin response to 1 unit of packed red blood cells, the blood warming method, the differences in fresh vs. old blood, the method of keeping fresh frozen plasma (FFP) and drugs for anemia. Each question had four answers, one was true and three were false. We assigned 1 point for a true answer and 0 points for a false one. The lowest and highest scores were 0 and 14, respectively. The levels of knowledge were defined as poor (0-7), intermediate: (8-10) and good (11-14). The third part of the questionnaire was related to the blood transfusion performance that we evaluated using the Likert scoring system with values of 0 (never) and 4 (always). The lowest score was 0 and the highest one was 40. The levels of performance were defined as low (0-30), intermediate (31-36) and good (37-40). The researcher held the educational course through the question-and-answer method and by delivering a presentation in 6 sessions to improve nurses’ knowledge and performance. The nurses were classified into three groups, two groups of 20 and a group of 15 individuals. In order to examine the association between the intervention and the nurses’ level of knowledge and performance, before and after the course we asked them to fill out the questionnaires. We analyzed data using SPSS 22, descriptive statistics such as frequency and percentage and analytical statistics such as Mann- Whitney, Wilcoxon, and Kruskal-Wallis. Results were considered significant if p<0.05.

## Results

Results showed that before the intervention the level of knowledge and performance for the majority of participants was poor (54%) and intermediate (64%), respectively. However, after the intervention, the level of knowledge and performance of the majority of participants reached the good level (72% and 88%, respectively). (Table 1)

Results indicated a significant difference between mean scores of knowledge and performance before and after the intervention (p=0.001). (Table 2)

The results showed that 54% of participants were women, 62% of nurses were younger than 30 years of age. 26% of nurses were working in the emergency department, 18% in gynecology and 16% in internal medicine and general surgery. After the intervention, the mean performance score of veteran nurses increased significantly (p<0.05). Before the intervention, we observed a significant association between mean score of performance, age and department. However, after the intervention, this association was not significant anymore (p>0.05). Sex was not significantly associated with nurses’ performance neither before nor after the intervention. Also, the association between nurses’ mean knowledge score and age, sex, work experience and department was not significant (p>0.05). (Table 3)

## Discussion

The present study aimed to evaluate the impact of an educational intervention on nurses’ level of knowledge and performance. According to the results, 54% of participants were women and all had a B.Sc. degree. In a study conducted by Silva and et al., more than 2/3 of participants were women with a B.Sc. degree [11]. In our study, before the educational intervention, most of the nurses had insufficient knowledge and intermediate performance. However, this intervention increased their knowledge and performance to a satisfactory level. In a Turkish study, the majority of nurses had intermediate performance and their knowledge scores were 50-100 [12] while a French study reported that nurses’ level of knowledge and performance about transfusion was poor. The nurses had knowledge about patients and appropriate blood products in 54% of cases. [13]. A study in Iran revealed that in medical care providers the level of knowledge about blood transfusion was poor in 26.2% of cases, intermediate in 22.1% and satisfactory in 51.6%. This study emphasized the importance of developing a guideline to improve personnel’ level of knowledge [14]. Findings of the present study showed a significant difference between knowledge and performance scores before and after the intervention. This finding was in agreement with other studies [15, 2]. Previous studies revealed that the effect of education on nurses’ performance was associated with other factors such as the method of education and their motivation to improve themselves [16, 17]. Regarding the important role of nurses in transfusion safety, it seems necessary to develop an appropriate educational program to improve their level of knowledge and performance [10, 14]. Similar to the results of other studies, our findings indicated a significant difference in the level of knowledge and performance in veteran nurses before and after the intervention [15, 18]. On the one hand, older nurses are more experienced in their jobs, but on the other hand, they had attended more educational courses about transfusion.

**Table 1:** Nurses’ level of knowledge and performance about blood and blood transfusion before and after the educational intervention

Variable		Level	Frequency (%)
Before intervention	Knowledge	Poor	27 (54)
		Intermediate	16 (32)
		Good	7 (14)
	Performance	Poor	5 (10)
		Intermediate	32 (64)
		Good	13 (26)
After intervention	Knowledge	Poor	1 (2)
		Intermediate	13 (26)
		Good	36 (72)
	Performance	Poor	0(0)
		Intermediate	6 (12)
		Good	44 (88)
Total	50		

**Table 2:** Comparison between nurses’ level of knowledge and performance about blood and blood transfusion before and after the educational intervention

Variable		Mean + SD	Min	max	P-value
Knowledge score	Before intervention	7.66 + 2.18	4	12	0.001
	After intervention	11.56 + 1.84	7	14	
Performance score	Before intervention	34.92 + 2.94	26	40	0.001
	After intervention	38.38 + 1.48	34	40	

**Table 3:** The association between socio-professional characteristics and knowledge and performance scores

variable		Frequency (%)	Knowledge score (p-value)	Performance score (p-value)
Sex	Female	27 (54)	0.60*	0.17*
	Male	23 (46)	0.44**	0.10**
Age (years)	<30	31 (62)	0.70*	0.002*
	30<	19 (38)	0.99**	0.70**
Working experience (years)	<6	33 (66)	0.14*	0.001*
	6<	17 (34)	0.39**	0.014**
Department	Emergency	13 (26)		
			0.44*	0.013*
			0.41**	0.42**
	Internal medicine	8 (16)		
	Dialysis	4 (8)		
	Gynecology	9 (18)		
	Neonates	4 (8)		
	General surgery	8 (16)		
	Operation room	2 (4)		
	Pediatrics	2 (4)		

* Before intervention

* After intervention

The results of the present study revealed that before the intervention, there was a significant association between age, department and nurses’ performance score. Nevertheless, after the intervention, this difference was not significant. Sex was not significantly associated with the performance score before and after the intervention. Studies by Watson et al. and Abdullahi and et al., showed no significant association between performance and sex, marital status, participation in educational courses and working experience [19, 20]. Blood transfusion is a lifesaving medical intervention, but it may become life-threatening if simplistically considered a basic medical procedure. In the medical care system, we aim to improve the effectiveness of blood transfusion. In concordance with other studies, our findings indicated that before and after the intervention, the association between nurses’ performance score and sex, age, working experience, department of occupation was not significant [21-26]. Lee and et al. reported a significant association between nurses’ performance score and their demographic characteristics (age, work experience and so forth) [26]. In several studies, it was indicated that nurses who attended educational courses were more knowledgeable about blood transfusion [13, 24, 25]. Regarding the important role of nurses in handling patients, it seems necessary to develop educational programs to improve their level of knowledge and practice.

## Conclusion

Educational programs regarding blood transfusion can positively impact on nurses’ level of knowledge and performance. All of the medical care personnel must be well educated about the appropriate condition of blood storage, injection and reaction to side effects of blood or blood products transfusion. It seems necessary to develop standard guidelines and educational courses about transfusion for nurses to improve their level of knowledge and practice.

## Conflict of Interest

The authors confirm that there are no conflicts of interest.
